# Association between delirium superimposed on dementia and mortality in hospitalized older adults: A prospective cohort study

**DOI:** 10.1371/journal.pmed.1002264

**Published:** 2017-03-28

**Authors:** Thiago J. Avelino-Silva, Flavia Campora, Jose A. E. Curiati, Wilson Jacob-Filho

**Affiliations:** Division of Geriatrics, Department of Internal Medicine, University of São Paulo Medical School, São Paulo, Brazil; University of California San Francisco Memory and Aging Center, UNITED STATES

## Abstract

**Background:**

Hospitalized older adults with preexisting dementia have increased risk of having delirium, but little is known regarding the effect of delirium superimposed on dementia (DSD) on the outcomes of these patients. Our aim was to investigate the association between DSD and hospital mortality and 12-mo mortality in hospitalized older adults.

**Methods and findings:**

This was a prospective cohort study completed in the geriatric ward of a university hospital in São Paulo, Brazil. We included 1,409 hospitalizations of acutely ill patients aged 60 y and over from January 2009 to June 2015.

Main variables and measures included dementia and dementia severity (Informant Questionnaire on Cognitive Decline in the Elderly, Clinical Dementia Rating) and delirium (Confusion Assessment Method). Primary outcomes were time to death in the hospital and time to death in 12 mo (for the discharged sample). Comprehensive geriatric assessment was performed at admission, and additional clinical data were documented upon death or discharge. Cases were categorized into four groups (no delirium or dementia, dementia alone, delirium alone, and DSD). The no delirium/dementia group was defined as the referent category for comparisons, and multivariate analyses were performed using Cox proportional hazards models adjusted for possible confounders (sociodemographic information, medical history and physical examination data, functional and nutritional status, polypharmacy, and laboratory covariates). Overall, 61% were women and 39% had dementia, with a mean age of 80 y. Dementia alone was observed in 13% of the cases, with delirium alone in 21% and DSD in 26% of the cases. In-hospital mortality was 8% for patients without delirium or dementia, 12% for patients with dementia alone, 29% for patients with delirium alone, and 32% for DSD patients (Pearson Chi-square = 112, *p <* 0.001). DSD and delirium alone were independently associated with in-hospital mortality, with respective hazard ratios (HRs) of 2.14 (95% CI = 1.33–3.45, *p =* 0.002) and 2.72 (95% CI = 1.77–4.18, *p <* 0.001). Dementia alone did not have a significant statistical association with in-hospital mortality (HR = 1.69, 95% CI = 0.72–2.30, *p =* 0.385). Finally, while 24% of the patients died after discharge, 12-mo mortality was not associated with dementia or delirium in any of the diagnostic groups (DSD: HR = 1.15, 95% CI = 0.79–1.68, *p =* 0.463; delirium alone: HR = 1.05, 95% CI = 0.71–1.54, *p =* 0.810; dementia alone: HR = 1.19, 95% CI = 0.79–1.78, *p =* 0.399). Limitations to this study include not exploring the effects of the duration and severity of delirium on the outcomes.

**Conclusions:**

DSD and delirium alone were independently associated with a worse prognosis in hospitalized older adults. Health care professionals should recognize the importance of delirium as a predictor of hospital mortality regardless of the coexistence with dementia.

## Abstract 2

In a prospective cohort study, Thiago Junqueira Avelino-Silva and colleagues examine the association of delirium superimposed on dementia on hospital mortality and 12-month mortality in hospitalized older adults.

## Author summary

**Why was this study done?:**

- Delirium is an acute disturbance of the mental state that frequently affects hospitalized patients, in particular older adults.
- Seriously ill older patients often have both delirium and dementia, but the prognostic effects of delirium superimposed on dementia (DSD) are still poorly understood.

**What did the researchers do and find?:**

- We followed a cohort of hospitalizations of acutely ill older adults admitted to the geriatric ward of a tertiary university hospital in São Paulo, Brazil, from January 2009 to June 2015.
- We included 1,409 admissions of patients with a mean age of 80 years.
- We confirmed the elevated frequency of delirium, dementia, and their coexistence in the acute care setting. We also observed high mortality rates in patients with DSD and delirium alone, both in the hospital and after discharge.
- After adjusting our analyses to account for possible confounding factors (sociodemographic information, medical history and physical examination data, functional and nutritional status, polypharmacy, and laboratory covariates), we found that DSD and delirium alone were predictive of a worse prognosis in the hospital setting, but not in the 12 months following discharge.

**What do these findings mean?:**

- Delirium is associated with poor short-term prognosis both in patients with and without preexisting dementia.
- Clinicians should be aware of this information and use it when discussing prognosis with patients and families/caregivers.

## Introduction

Delirium is an acute disturbance of the mental state that has a fluctuating nature and is characterized by inattention and cognitive impairment. Its occurrence depends on an intricate relationship between predisposing and precipitating factors, which amount to more than 60 characteristics associated with delirium [1]. Older adults who have three or more of these characteristics have a 60% higher risk of developing delirium. Advanced age, preexisting dementia or cognitive impairment, functional dependence, and visual impairment are particularly relevant risk factors in this context [2].

Coexistence between delirium and dementia is highly frequent, and hospitalized patients with dementia are up to eight times more likely to have delirium [3]. Compared with isolated dementia, having both delirium and dementia is associated with higher costs, more pronounced functional decline, and increased mortality [4,5]. Fick et al. studied 139 older adults with dementia admitted to a community hospital; they found an incidence of delirium of 32% and demonstrated an increase in the length of hospital stay in these cases [4].

Despite the high prevalence of delirium and dementia in the hospital setting, evidence regarding the effects of their coexistence on outcomes in older adults is still limited and conflicting [3]. For instance, one study found that hospitalized patients with delirium superimposed on dementia (DSD) were two times as likely to die in their 2-y follow-up [6]. Conversely, another study reported that 12-mo mortality was highest in older adults with delirium and without dementia [7]. Therefore, our aim was to investigate the association between DSD and hospital mortality and 12-mo mortality in a large cohort of hospitalized older adults, with the hypothesis that DSD will be associated with increased mortality.

## Methods

### Ethical considerations

This study was approved by the local institutional review board (Comissão de Ética para Análise de Projetos de Pesquisa do Hospital das Clínicas da Faculdade de Medicina de Universidade São Paulo). Written consent was obtained from participants, and principles expressed in the Declaration of Helsinki were respected. All patient-identifiable information was stored in locked cabinets and/or secure electronic servers.

### Study design and population

We followed a cohort of acutely ill patients admitted to a geriatric ward of a tertiary university hospital in São Paulo, Brazil. The unit admits medical patients aged 60 y and over and is staffed with a multidisciplinary team that includes geriatricians, nurses, physiotherapists, speech therapists, social workers, psychologists, and nutritionists. A minimum age of 60 y is the only specific requirement for admittance in the ward, but older adults with high clinical complexity and vulnerability are preferentially referred for admission.

We included consecutive hospitalizations from January 2009 to June 2015 with the following criteria: (1) age of 60 y or over and (2) admission for acute illness (defined as disease of recent onset, or as recent complication of chronic disease, requiring hospitalization for clinical management). Cases were excluded based on the following criteria: (1) admission for end-of-life care, (2) incomplete data on the main variables, (2) length of stay shorter than 48 h, and (4) patient or caregiver refusal to authorize use of hospital data for research.

### Measures

#### Outcomes

This study is part of a broad project designed to explore the prognostic significance of several aspects of delirium. The primary endpoints for this particular study were (1) time to death during hospitalization, analyzed for the whole sample, and (2) time to death in 12 mo, analyzed for participants who were discharged from the hospital.

Time to death in the hospital was documented at the end of hospitalization. Time to death in the follow-up period was obtained by one of the authors (T. J. A.-S.), who contacted patients 12 mo after admission. Up to three telephone contact attempts were made on different days of the week, including Saturdays. When unable to reach patients or caregivers, T. J. A.-S. examined medical records (including medical consultations, consultations with the geriatric ward multidisciplinary team, and records of tests and procedures) to identify the last date on which the participant was known to be alive. Non-respondents for whom follow-up medical records did not reach the end of the follow-up period were considered lost to follow-up and dealt accordingly in the statistical analysis.

#### Predictors

Patients were evaluated according to a standardized comprehensive geriatric assessment that is consistently performed within the first 24 h of admission [8]. Supplementary information regarding new diagnoses and complications, functional status, and hospitalization summary were recorded at discharge or death. Geriatric fellows were trained by T. J. A.-S. in up to four 60-min sessions to perform the assessments, which included detailed cognitive evaluation. T. J. A.-S. was available throughout their rotations to clarify any lingering questions. The assessments were also supervised and adjudicated by two attending geriatricians who had up to 30 y of experience in caring for acutely ill older adults and who participated in the development of the comprehensive geriatric assessment model. Study data were collected and managed using REDCap electronic data capture tools [9].

Delirium was detected using the short form of the Confusion Assessment Method (CAM) [10], both at admission and daily throughout the hospital stay. The application of the short CAM was standardized per its developers’ training manual [10]. Delirium severity was measured using the Delirium Index, which assesses seven domains of the long CAM (attention, thinking, level of consciousness, orientation, memory, sensory perception, and psychomotor activity) and generates scores ranging from 0 to 21 (21 = most severe) [11]. Cognitive status was determined using the Mini-Mental State Exam, Clinical Dementia Rating (CDR), and the short form of the Informant Questionnaire on Cognitive Decline in the Elderly (IQCODE) [12,13]. CDR and IQCODE were adapted to compare the patient’s status 10 y before hospitalization with characteristics 3 mo before hospitalization, to exempt the evaluation from being distorted by clinical conditions at admission. Dementia was defined by a CDR score of 1 or more and an IQCODE score above 3.38. The primary independent variable was a composite of delirium and dementia diagnosis, and we examined the overlap by categorizing the cases into four groups: no delirium or dementia, dementia alone, delirium alone, and DSD. Since information provided by relatives and caregivers is crucial to the definition of cognitive impairment, examiners were instructed to interview informants with the closest relationship possible to the patients.

Covariates of interest included the following: sociodemographic information (age, sex, marital status, literacy, economic classification, and referring unit); medical history and physical examination data (comorbidities, visual and auditory deficits, vital signs, and admission diagnoses); functional status (six activities of daily living; each activity was scored on a scale ranging from 0 to 2 points, and a final score was generated from the total sum of the items; range = 0–12, 12 = best) [14]; nutritional status (Mini Nutritional Assessment) [15]; polypharmacy (defined as the chronic use of five or more medications) [16]; and laboratory tests (hemoglobin, total leukocytes, C-reactive protein, glomerular filtration rate [GFR], urea, sodium, potassium, sodium bicarbonate, albumin, and 25-hydroxyvitamin D).

### Statistical analysis

The analysis plan for this study was developed as part of a doctorate thesis investigating prognostic factors in acutely ill older adults with delirium and was determined beforehand, during the designing stages of the study. The analysis did not differ from the original plan.

A descriptive analysis of demographic, clinical, and laboratory characteristics was performed using counts and proportions, means and standard deviations, and medians and interquartile ranges. Categorical variables were compared using the Chi-square test or Fisher’s exact test as appropriate. Continuous variables were compared using one-way ANOVA or the Kruskal–Wallis test as appropriate.

We defined the date of the diagnosis of delirium as time zero for the survival analyses. Kaplan–Meier curves were used to represent unadjusted hospital survival and 12-mo survival according to delirium and dementia diagnosis, and log-rank tests to compare the groups. The association between DSD and time to death was analyzed using Cox proportional hazards models adjusted for the following preselected covariates: age, sex, marital status, referring unit, functional status (activities of daily living), nutritional status, comorbidities (hypertension, diabetes, heart failure, cerebrovascular disease, coronary disease, chronic obstructive pulmonary disease, and cancer), polypharmacy, vital signs (heart rate and mean arterial pressure), GFR, urea, albumin, total leucocytes, and C-reactive protein. A sensitivity analysis was performed grouping patients with mild dementia with those without dementia, based on the hypothesis that these patients might follow more similar clinical paths. The analyses were performed within patients, considering that each individual might have had more than one hospitalization during the study period. All statistical tests were two-tailed, and an alpha error of up to 5% was accepted. Statistical analyses were performed using Stata SE 14.1 (StataCorp).

## Results

**Fig 1:**
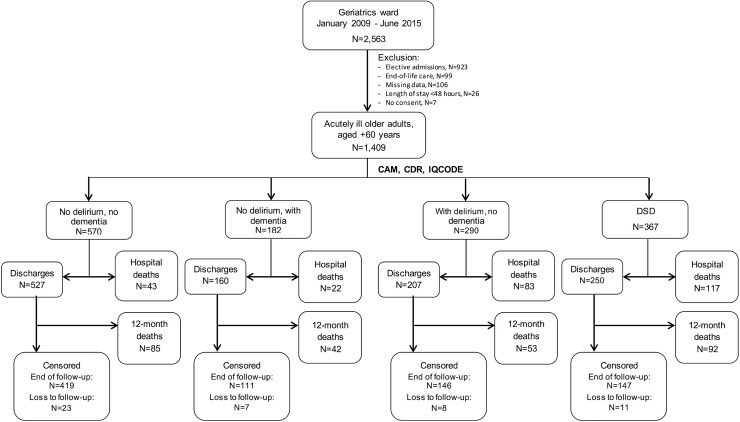
Flowchart of inclusion and follow-up in the study. A total of 1,409 admissions from January 2009 to June 2015 were included in the study, with 367 cases of DSD. Dementia alone was identified in 182 admissions, and delirium alone in 290. Individuals with missing data represented only a minor proportion of the sample and were excluded from the analysis. CAM, Confusion Assessment Method; CDR, Clinical Dementia Rating; DSD, delirium superimposed on dementia; IQCODE, Informant Questionnaire on Cognitive Decline in the Elderly.

We included 1,409 hospitalizations, representing 1,204 patients (Fig 1). Participants were predominantly very old, female, and from middle- to low-income groups (Table 1). Most admissions were referred from the emergency department, and nearly half of these cases waited at least 48 h before being transferred to our unit. Median length of hospital stay was of 15 d (interquartile range [IQR] = 9; 26). The median Charlson Comorbidity Index score was 3 (IQR = 1; 5).

**Table 1:** Characteristics of acutely ill hospitalized older adults, according to delirium and dementia diagnosis, 2009–2015.

Characteristic	Total, *n =* 1,409	No delirium/dementia, *n =* 570 (40%)	Dementia alone, *n =* 182 (13%)	Delirium alone, *n =* 290 (21%)	Delirium superimposed on dementia, *n =* 367 (26%)	*p*-Value
**Age (years), mean (SD)**	80 (± 9)	78 (± 9)	82 (± 8)	80 (± 9)	84 (± 8)	<0.001
**Age (years)**						
60–69	175 (12)	114 (20)	13 (7)	35 (12)	13 (4)	<0.001
70–79	440 (31)	211 (37)	53 (29)	95 (33)	81 (22)	
80–89	558 (40)	184 (32)	83 (46)	118 (41)	173 (47)	
≥90	236 (17)	61 (11)	33 (18)	42 (14)	100 (27)	
**Female**	860 (61)	325 (57)	126 (69)	180 (62)	229 (62)	0.024
**Marital status**						
Married	539 (38)	240 (42)	73 (40)	109 (38)	117 (32)	<0.001
Widowed	657 (47)	230 (40)	92 (51)	130 (45)	205 (56)	
Single	124 (9)	51 (9)	11 (6)	32 (11)	30 (8)	
Divorced/separated	89 (6)	49 (9)	6 (3)	19 (7)	15 (4)	
**Referring unit**						
Outpatient clinic	536 (38)	258 (45)	100 (55)	68 (23)	110 (30)	<0.001
Emergency department	772 (55)	274 (48)	77 (42)	193 (67)	228 (62)	
Intensive care unit	101 (7)	38 (7)	5 (3)	29 (10)	29 (8)	
**Polypharmacy**	851 (60)	346 (61)	121 (66)	175 (60)	209 (57)	0.198
**Admission functional status (ADL points)**						
9–12	485 (34)	355 (62)	47 (26)	60 (21)	23 (6)	<0.001
5–8	315 (22)	139 (24)	48 (26)	79 (27)	49 (13)	
0–4	609 (43)	76 (13)	87 (48)	151 (52)	295 (80)	
**Mini Nutritional Assessment**						
Normal	213 (15)	147 (26)	19 (10)	38 (13)	9 (2)	<0.001
Risk of malnutrition	595 (42)	286 (50)	76 (42)	135 (47)	98 (27)	
Malnutrition	601 (43)	137 (24)	87 (48)	117 (40)	260 (71)	
**Comorbidities**						
Hypertension	1,020 (72)	411 (72)	135 (74)	213 (73)	261 (71)	0.856
Diabetes	448 (32)	169 (30)	60 (33)	98 (34)	121 (33)	0.553
Heart failure	389 (28)	161 (28)	55 (30)	77 (27)	96 (26)	0.733
Cerebrovascular disease	286 (20)	61 (11)	60 (33)	47 (16)	116 (32)	<0.001
Coronary disease	246 (17)	103 (18)	35 (19)	51 (18)	57 (16)	0.684
COPD	172 (12)	75 (13)	25 (14)	34 (12)	38 (10)	0.549
Cancer	154 (11)	71 (12)	15 (8)	36 (12)	32 (9)	0.157
Depression	313 (22)	106 (19)	56 (31)	61 (21)	90 (25)	0.004
**Heart rate (bpm)**						
<60	61 (4)	30 (5)	8 (4)	8 (3)	15 (4)	0.439
60–100	1,248 (89)	505 (89)	164 (90)	258 (89)	321 (88)	
≥100	100 (7)	35 (6)	10 (5)	24 (8)	31 (8)	
**Mean arterial pressure < 90 mm Hg**	719 (51)	283 (50)	97 (53)	146 (50)	193 (53)	0.748
**Leucocytes ≥ 11 × 10**^**3**^ **cells/mm**^**3**^	321 (23)	108 (19)	35 (19)	83 (29)	95 (26)	0.003
**Albumin < 33 g/l**	843 (60)	292 (51)	101 (55)	191 (66)	259 (71)	<0.001
**GFR (ml/min)**						
≥60	705 (50)	284 (50)	98 (54)	134 (46)	189 (52)	0.054
30–59	492 (35)	212 (37)	63 (35)	96 (33)	121 (33)	
<30	212 (15)	74 (13)	21 (12)	60 (21)	57 (16)	
**Urea ≥ 3.3 mmol/l**	328 (23)	110 (19)	39 (21)	78 (27)	101 (28)	0.011
**C-reactive protein > 476 nmol/l**	560 (40)	194 (34)	60 (33)	120 (41)	186 (51)	<0.001

Data given as *n* (percent) unless otherwise indicated.

ADL, activities of daily living; bpm, beats per minute; COPD, chronic obstructive pulmonary disease; GFR, glomerular filtration rate; SD, standard deviation.

Only 15% of the patients could be classified as having normal nutritional status according to the Mini Nutritional Assessment (median score = 8; IQR = 5; 11). Other common geriatric syndromes in our sample were depression, urinary incontinence, falls, sensory deficits, and pressure ulcers. In the initial laboratory assessment, the mean value of serum albumin was low (32 ± 06 g/l), and the median value of C-reactive protein was high (352 nmol/l; IQR = 105; 838). Mean hemoglobin level at admission was 110 (± 23) g/l, and mean GFR was 67 (± 39) ml/min.

We identified delirium at admission in 379 (27%) hospitalizations. We subsequently detected delirium in the course of an additional 278 (20%) hospitalizations. Dementia was present in 549 (39%) admissions. Alzheimer disease was observed in 38% of the cases of dementia, vascular dementia in 23%, mixed dementia in 20%, and dementia from other causes in 19%. The median score on the short IQCODE was 3.63 (IQR = 3.13; 4.56). DSD was verified in 367 (26%) hospitalizations, and 254 (69%) of these patients had moderate to severe dementia. Hypoactive delirium was the most frequently observed motor subtype in both delirium alone and DSD cases (51% and 55%, respectively). The median Delirium Index score upon delirium diagnosis was 14 (IQR = 11; 17) in DSD cases and 11 (IQR = 8; 15) in delirium alone cases (Kruskal–Wallis test, *p <* 0.001). The median length of hospital stay for the no delirium/dementia, dementia alone, delirium alone, and DSD groups was, respectively, 14 d (IQR = 8; 22), 13 d (IQR = 8; 20), 19 d (IQR = 10; 31), and 17 d (IQR = 9; 31) (Kruskal–Wallis test, *p <* 0.001).

**Fig 2:**
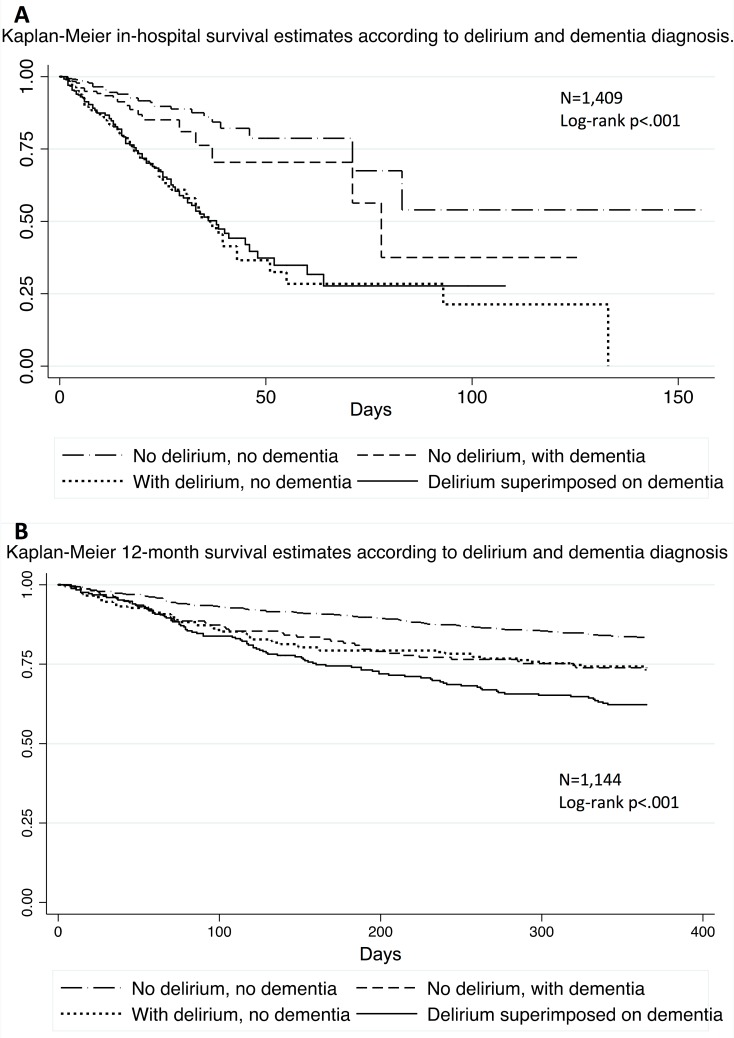
Probability of survival according to delirium and dementia diagnosis. (A) Kaplan–Meier estimates representing the probability of survival in the hospital according to delirium and dementia diagnosis, with a corresponding log-rank test indicating a statistically significant difference between the groups. (B) Kaplan–Meier estimates representing the probability of survival over 12 mo according to delirium and dementia diagnosis, with a corresponding log-rank test indicating a statistically significant difference between the groups.

A total of 265 (19%) older adults died in the hospital, and an additional 272 died in the follow-up period after discharge. Examining hospital mortality according to delirium and dementia diagnosis, we found that mortality was 8%, 12%, 29%, and 32% for the no delirium/dementia, dementia alone, delirium alone, and DSD groups, respectively (Pearson Chi-square = 112, *p <* 0.001). After discharge, 12-mo mortality was 37% in the DSD group, compared to 16% in the group without delirium or dementia, 26% in the dementia alone group, and 26% in the delirium alone group (Pearson Chi-square = 41, *p <* 0.001). We found the differences between the unadjusted mortality probabilities according to delirium and dementia diagnosis to be statistically significant (log-rank tests, *p <* 0.001) (Fig 2).

Compared with no delirium/dementia, delirium alone and DSD were associated with greater risk for hospital mortality after adjustment for several possible confounders (age, sex, marital status, referring unit, functional status, nutritional status, comorbidities, polypharmacy, vital signs, GFR, urea, albumin, total leucocytes, and C-reactive protein; Table 2). We did not observe a statistically significant association between dementia alone and hospital mortality. Delirium alone was particularly predictive of in-hospital death, with a hazard ratio (HR) of 2.72 (95% CI = 1.77–4.18, *p* < 0.001), compared to 2.14 (95% CI = 1.33–3.45, *p =* 0.002) for DSD and 1.29 (95% CI = 0.72–2.30, *p =* 0.385) for dementia alone. In \the adjusted analyses, we could not demonstrate an independent association between delirium, dementia, or DSD and 12-mo mortality (Table 3).

**Table 2:** Association between delirium superimposed on dementia and hospital mortality in acutely ill older adults, 2009–2015 (*n* = 1,409 admissions/1,204 patients).

Variable	Hospital mortality, *n* (percent)	Bivariate hazard ratio (95% CI)	Adjusted hazard ratio^a^ (95% CI)	Adjusted *p-*value
**Delirium/dementia**				
No delirium/dementia	43 (8)	Ref.	Ref.	Ref.
Dementia only	22 (12)	1.69 (1.02–2.80)	1.29 (0.72–2.30)	0.385
Delirium only	83 (29)	3.93 (2.72–5.68)	2.72 (1.77–4.18)	<0.001
Delirium superimposed on dementia	117 (32)	3.82 (2.69–5.43)	2.14 (1.33–3.45)	0.002
**Age (10-y increase)**^b^	—	1.44 (1.25–1.66)	1.24 (1.03–1.49)	0.021
**Sex**				
Male	97 (18)	Ref.	Ref.	Ref.
Female	168 (20)	1.18 (0.92–1.51)	1.09 (0.80–1.48)	0.588
**Marital status**				
Married	88 (16)	Ref.	Ref.	Ref.
Widowed	138 (21)	1.31 (1.01–1.72)	0.79 (0.56–1.12)	0.178
Single	22 (18)	1.15 (0.72–1.84)	1.04 (0.64–1.68)	0.791
Divorced/separated	17 (19)	0.98 (0.58–1.66)	0.74 (0.41–1.37)	0.343
**Referring unit**				
Outpatient clinic	79 (15)	Ref.	Ref.	Ref.
Emergency department	171 (22)	1.36 (1.04–1.78)	1.36 (1.03–1.79)	0.032
Intensive care unit	15 (15)	0.95 (0.55–1.66)	0.77 (0.58–1.01)	0.059
**Admission functional status (ADL points)**				
9–12	37 (8)	Ref.	Ref.	Ref.
5–8	43 (14)	1.67 (1.08–2.59)	0.97 (0.61–1.56)	0.905
0–4	185 (30)	3.14 (2.20–4.48)	1.21 (0.80–1.85)	0.367
**Mini Nutritional Assessment**				
Normal	14 (7)	Ref.	Ref.	Ref.
Risk of malnutrition	65 (11)	1.41 (0.79–2.52)	1.05 (0.53–2.08)	0.881
Malnutrition	186 (31)	3.64 (2.11–6.27)	2.28 (1.15–4.53)	0.019
**Polypharmacy**				
No	129 (23)	Ref.	Ref.	Ref.
Yes	136 (16)	0.76 (0.60–0.97)	0.77 (0.58–1.01)	0.059
**Cerebrovascular disease**				
No	210 (19)	Ref.	Ref.	Ref.
Yes	55 (19)	1.04 (0.78–1.41)	0.81 (0.57–1.16)	0.255
**Depression**				
No	202 (18)	Ref.	Ref.	Ref.
Yes	63 (20)	1.21 (0.91–1.60)	1.24 (0.89–1.72)	0.206
**Diabetes mellitus**				
No	179 (19)	Ref.	Ref.	Ref.
Yes	86 (19)	1.12 (0.87–1.45)	1.34 (0.99–1.83)	0.062
**Coronary disease**				
No	218 (19)	Ref.	Ref.	Ref.
Yes	47 (19)	1.05 (0.77–1.44)	0.90 (0.61–1.32)	0.580
**Heart failure**				
No	186 (18)	Ref.	Ref.	Ref.
Yes	79 (20)	1.17 (0.90–1.52)	1.28 (0.91–1.83)	0.157
**COPD**				
No	227 (18)	Ref.	Ref.	Ref.
Yes	38 (22)	1.29 (0.91–1.82)	1.20 (0.84–1.72)	0.318
**Cancer**				
No	212 (17)	Ref.	Ref.	Ref.
Yes	53 (34)	1.83 (1.35–2.47)	1.73 (1.21–2.47)	0.002
**Infection (at admission)**				
No	110 (15)	Ref.	Ref.	Ref.
Yes	155 (23)	1.35 (1.05–1.72)	0.78 (0.59–1.04)	0.086
**Heart rate (bpm)**				
60–100	230 (18)	Ref.	Ref.	Ref.
<60	8 (13)	0.58 (0.29–1.17)	0.67 (0.32–1.40)	0.274
≥100	27 (27)	1.36 (0.91–2.03)	1.07 (0.68–1.68)	0.773
**Mean arterial pressure (mm Hg)**				
≥90	110 (16)	Ref.	Ref.	Ref.
<90	155 (22)	1.26 (0.99–1.61)	1.12 (0.86–1.45)	0.398
**Albumin (g/l)**	—	0.55 (0.46–0.67)	0.62 (0.49–0.80)	<0.001
**GFR (ml/min)**				
≥60	115 (16)	Ref.	Ref.	Ref.
30–59	93 (19)	1.35 (1.03–1.78)	1.53 (1.15–2.03)	0.003
<30	57 (27)	1.75 (1.27–2.41)	1.56 (1.04–2.32)	0.030
**Urea (mmol/l)**				
<3.3	178 (16)	Ref.	Ref.	Ref.
≥3.3	87 (27)	1.52 (1.17–1.96)	1.10 (0.80–1.51)	0.555
**Leucocytes (10**^**3**^ **cells/l)**				
<11	173 (16)	Ref.	Ref.	Ref.
≥11	92 (29)	1.70 (1.32–2.20)	1.32 (0.99–1.78)	0.061
**C-reactive protein (nmol/l)**				
≤476	114 (13)	Ref.	Ref.	Ref.
>476	151 (27)	1.64 (1.28–2.10)	1.23 (0.93–1.62)	0.150

^a^Cox proportional hazards model adjusted for all the variables included in the table.

^b^Age was analyzed as a continuous variable, and results indicate estimates for each additional decade of life.

ADL, activities of daily living; bpm, beats per minute; COPD, chronic obstructive pulmonary disease; GFR, glomerular filtration rate; Ref., referent category.

**Table 3:** Association between delirium superimposed on dementia and 12-mo mortality in acutely ill older adults; 2009–2015 (*n* = 1,144 discharges/976 patients).

Variable	12-mo mortality, *n* (percent)	Bivariate hazard ratio (95% CI)	Adjusted hazard ratio^a^ (95% CI)	Adjusted *p*-value
**Delirium/dementia**				
No delirium/dementia	85 (16)	Ref.	Ref.	Ref.
Dementia only	42 (26)	1.75 (1.16–2.63)	1.19 (0.79–1.78)	0.399
Delirium only	53 (26)	1.73 (1.21–2.47)	1.05 (0.71–1.54)	0.810
Delirium superimposed on dementia	92 (37)	2.60 (1.91–3.55)	1.15 (0.79–1.68)	0.463
**Age (10-y increase)**^b^	—	1.32 (1.12–1.56)	1.27 (1.05–1.54)	0.012
**Sex**				
Male	113 (25)	Ref.	Ref.	Ref.
Female	159 (23)	0.90 (0.69–1.17)	0.93 (0.68–1.26)	0.632
**Marital status**				
Married	115 (26)	Ref.	Ref.	Ref.
Widowed	126 (24)	0.95 (0.31–0.91)	0.77 (0.55–1.08)	0.126
Single	15 (15)	0.53 (0.73–1.25)	0.54 (0.32–0.92)	0.023
Divorced/separated	16 (22)	0.81 (0.48–1.39)	0.95 (0.54–1.67)	0.846
**Referring unit**				
Outpatient clinic	98 (21)	Ref.	Ref.	Ref.
Emergency department	140 (23)	1.11 (0.86–1.45)	0.90 (0.68–1.20)	0.484
Intensive care unit	34 (40)	2.19 (1.46–3.29)	1.83 (1.16–2.89)	0.010
**Admission functional status (ADL points)**				
9–12	60 (13)	Ref.	Ref.	Ref.
5–8	56 (21)	1.62 (1.13–2.33)	1.45 (1.00–2.10)	0.050
0–4	156 (37)	3.24 (2.37–4.43)	2.48 (1.75–3.52)	<0.001
**Mini Nutritional Assessment**				
Normal	33 (17)	Ref.	Ref.	Ref.
Risk of malnutrition	109 (21)	1.28 (0.86–1.90)	0.96 (0.64–1.45)	0.847
Malnutrition	130 (31)	2.07 (1.38–3.10)	1.17 (0.74–1.83)	0.501
**Polypharmacy**				
No	109 (25)	Ref.	Ref.	Ref.
Yes	163 (21)	0.87 (0.68–1.12)	0.93 (0.70–1.23)	0.603
**Cerebrovascular disease**				
No	203 (22)	Ref.	Ref.	Ref.
Yes	69 (30)	1.40 (1.05–1.86)	1.25 (0.90–1.75)	0.183
**Depression**				
No	211 (24)	Ref.	Ref.	Ref.
Yes	61 (24)	1.02 (0.76–1.37)	1.05 (0.76–1.45)	0.773
**Diabetes mellitus**				
No	191 (24)	Ref.	Ref.	Ref.
Yes	81 (22)	0.90 (0.68–1.19)	0.94 (0.69–1.28)	0.695
**Coronary disease**				
No	235 (25)	Ref.	Ref.	Ref.
Yes	37 (19)	0.73 (0.52–1.04)	0.66 (0.44–1.00)	0.048
**Heart failure**				
No	199 (24)	Ref.	Ref.	Ref.
Yes	73 (24)	0.98 (0.74–1.29)	1.21 (0.87–1.67)	0.259
**COPD**				
No	239 (24)	Ref.	Ref.	Ref.
Yes	33 (25)	1.07 (0.73–1.56)	1.20 (0.82–1.75)	0.356
**Cancer**				
No	247 (24)	Ref.	Ref.	Ref.
Yes	25 (25)	1.13 (0.74–1.74)	1.18 (0.73–1.90)	0.505
**Infection (at admission)**				
No	121 (19)	Ref.	Ref.	Ref.
Yes	151 (30)	1.59 (1.24–2.03)	1.26 (0.95–1.67)	0.108
**Heart rate (bpm)**				
60–100	240 (24)	Ref.	Ref.	Ref.
<60	11 (21)	0.85 (0.47–1.54)	1.01 (0.55–1.86)	0.976
≥100	21 (29)	1.25 (0.81–1.92)	1.00 (0.61–1.65)	0.987
**Mean arterial pressure (mm Hg)**				
≥90	136 (23)	Ref.	Ref.	Ref.
<90	136 (24)	1.02 (0.80–1.29)	1.05 (0.82–1.34)	0.681
**Albumin (g/l)**	—	0.70 (0.58–0.83)	0.87 (0.71–1.07)	0.186
**GFR (ml/min)**				
≥60	127 (22)	Ref.	Ref.	Ref.
30–59	101 (25)	1.21 (0.93–1.57)	1.42 (1.07–1.87)	0.015
<30	44 (28)	1.39 (0.99–1.96)	1.58 (1.07–2.34)	0.023
**Urea (mmol/l)**				
<3.3	210 (23)	Ref.	Ref.	Ref.
≥3.3	62 (26)	1.14 (0.86–1.51)	0.76 (0.54–1.06)	0.111
**Leucocytes (10**^**3**^ **cells/mm**^**3**^**)**				
<11	210 (23)	Ref.	Ref.	Ref.
≥11	62 (27)	1.20 (0.91–1.60)	0.98 (0.72–1.31)	0.870
**C-reactive protein (nmol/l)**				
≤476	153 (21)	Ref.	Ref.	Ref.
>476	119 (29)	1.53 (1.20–1.95)	1.34 (1.03–1.72)	0.027

^a^Cox proportional hazards model adjusted for all the variables included in the table.

^b^Age was analyzed as a continuous variable, and results indicate estimates for each additional decade of life.

ADL, activities of daily living; bpm, beats per minute; COPD, chronic obstructive pulmonary disease; GFR, glomerular filtration rate; Ref., referent category.

A sensitivity analysis grouping cases of mild dementia with those without cognitive impairment did not produce substantially different results from the main analysis regarding in-hospital death. Compared with no delirium/dementia, the adjusted HRs for hospital mortality in the dementia alone, delirium alone, and DSD groups were, respectively, 1.68 (95% CI = 0.90–3.15, *p =* 0.104), 2.65 (95% CI = 1.77–3.95, *p <* 0.001), and 2.36 (95% CI = 1.48–3.78, *p <* 0.001). However, the adjusted HRs for 12-mo mortality in the dementia alone, delirium alone, and DSD groups were, respectively, 1.83 (95% CI = 1.15–2.93, *p =* 0.012), 1.23 (95% CI = 0.88–1.72, *p =* 0.221), and 1.26 (95% CI = 0.84–1.89, *p =* 0.267). These results indicate that cases of moderate to severe dementia in the absence of delirium were associated with increased 12-mo mortality.

## Discussion

In our cohort of acutely ill hospitalized older adults, we observed that DSD occurred in 26% of admissions. Approximately one in three of these admissions resulted in death during hospitalization, with a cumulative mortality of 57% at 12 mo. The high frequency of delirium and dementia and the elevated mortality rates are consistent with data reported in the literature [2,17]. We found that delirium in the absence of dementia and DSD were both associated with increased hospital mortality compared to those with no delirium/dementia. In contrast, we did not find statistically significant associations between delirium, dementia, or DSD and 12-mo mortality after adjustment for confounding factors. Other factors demonstrated to have prognostic importance in our cohort were age, admission from an emergency department or intensive care unit, functional dependency, malnutrition, cancer, reduced GFR, and hypoalbuminemia.

Our results confirm that overlap between delirium and dementia is common. Dementia represents an important risk factor for delirium, and delirium has been increasingly linked with long-term cognitive decline [2]. However, evidence on how the combined effects of these conditions influence clinical outcomes is conflicting.

In a prospective cohort of hospitalized older adults, McCusker et al. reported that the occurrence of delirium was an independent predictor of mortality that was particularly strong among individuals without preexisting dementia (HR = 3.77, 95% CI = 1.39–10.20) [7]. Yet they did not find a statistically significant association between delirium and mortality in individuals with dementia (HR = 1.96, 95% CI = 0.76–5.05). In a sample of 425 patients aged 70 y or more from geriatric wards and long-term institutions, Laurila et al. also showed lower survival rates at 12 mo for the group with delirium and without dementia (HR = 2.10, 95% CI = 1.16–3.77) but not for the group with DSD (HR = 1.24, 95% CI = 0.64–2.42) [18]. The analysis was adjusted for age, sex, comorbidities, and dementia severity, but it should be noted that it was not planned in the original study design [19].

In contrast, Francis and Kapoor studied 229 patients aged 70 y or more admitted to a general hospital and concluded that the higher mortality observed in older patients with delirium was largely explained by the existence of previous functional and cognitive impairment [6]. The authors cited as limitations of their findings that the sample included only 46 cases of delirium and that institutionalized patients and those with advanced dementia were excluded. Similar results were published by Bellelli et al., who compared four paired groups of 47 older patients admitted to a rehabilitation center (without delirium or dementia, dementia alone, delirium alone, and DSD) and found that DSD cases had a significantly higher risk of death in 12 mo than any of the other three groups [20]. Still, the sample size and specificity of the study setting were potential limitations of the work. In a subsequent study in the same center, Morandi et al. confirmed the association between DSD and an increased risk of mortality over 12 mo (odds ratio = 1.8, 95% CI = 1.1–2.8) [5].

We found that delirium was associated with a greater risk of hospital mortality in the presence of dementia, but was exceptionally so in the absence of preexisting cognitive decline. As we have seen, this finding is consistent with descriptions of other studies, but it is still controversial. Delirium has been largely accepted as a predictor of mortality in hospitalized older adults [2], but the reasons behind diverging results when dementia comes into play remain to be explained.

One possible explanation for the discrepancy is that it may be more difficult to diagnose delirium in the context of dementia [21], resulting in misclassifications and errors in analysis. This might explain why, in our sensitivity analysis, we found an association between moderate and severe dementia and 12-mo mortality but not between DSD and the same outcome. Presuming that it is more difficult to diagnose delirium in these stages of dementia, one must accept the possibility that in some cases behavioral symptoms of dementia might have been wrongly defined as delirium, and vice versa, generating biases in the results.

Another possibility is that the prognostic significance of delirium in the presence of dementia is indeed different. It could be argued, for example, that the intensity of insult needed to trigger delirium in patients with impaired cognitive reserve is less than that required among those who do not have a cognitive deficit. Consequently, delirium might represent a less severe clinical complication in patients with dementia [7]. This could explain why we found that those with delirium alone had the greatest risk of hospital mortality, and why some studies did not demonstrate an association of DSD with worse prognosis.

Our study had limitations. It was conducted in a single center, in a unit specialized in the care of geriatric patients with high clinical complexity and vulnerability. Although many of our results are consistent with those described by other groups, these aspects of the study may have decreased its external validity. The prolonged hospital stays and elevated mortality are of note, but it is likely that these are the product not only of a specialized setting, but also of a health care system inefficient in providing high-quality primary care and effective post-hospital rehabilitation. The elevated hospital mortality we observed also raises the concern of immortal time bias in this study. If a substantial number of patients died before having the chance of developing delirium, its association with our outcomes might have been weakened.

We examined the association of the occurrence of delirium and dementia with mortality, adjusting our analysis for numerous clinical variables that have rarely been controlled for in the context of hospitalized older adults. Nevertheless, it is reasonable to assume that there are predictors of mortality in this population that were not measured or contemplated. The duration of delirium episodes and dementia etiologies are some of the aspects we did not investigate. We also did not explore the effect of the overall severity of delirium symptoms on prognosis, though DSD cases had higher Delirium Index scores upon delirium diagnosis.

It is possible that a greater proportion of non-respondents in the 12-mo follow-up had delirium and/or dementia as compared to respondents, which would introduce bias into the analyses, pulling the results towards the null. However, we believe that because the number of dropouts in the study was small (less than 8% in all four groups), this effect on our findings should have been minimum. Finally, the involvement of multiple geriatric fellows as study examiners may have affected our measures. Although we did not establish inter-rater reliability estimates, we point out that the examiners were routinely trained by the same investigator and supervised by the same clinicians to confer consistency to the method.

Our work also had several positive aspects. This is one of the largest cohorts ever to be analyzed to investigate the effects of DSD on the mortality of acutely ill older adults. Data were recorded prospectively and systematically, following a standardized model of comprehensive geriatric assessment, which allowed for a detailed view of the health of the study participants. In addition, our routines ensured a horizontal follow-up of all admitted patients, with daily discussions with experienced geriatricians, reducing the risk of non-detection of delirium, dementia, or other geriatric syndromes.

In summary, delirium was associated with poor prognosis in patients both with and without preexisting dementia in our cohort of acutely ill older adults. However, it is possible that DSD has different outcome implications compared to delirium alone, and that strategies to prevent and treat delirium in persons with dementia might need specific approaches. Inouye et al., in their classic study on a multicomponent intervention to prevent delirium in hospitalized older patients, found that those with dementia were possibly more responsive to measures such as orientation, therapeutic activities, mobility activities, and avoidance of psychotropic medications [22]. The same can be said of the clinical and prognostic meaning of delirium in the absence of previous cognitive impairment. Clinicians should be aware of the repercussions of this diagnosis and use it as one of the foundations from which to discuss prognosis with patients and families/caregivers. Future studies should focus on individualized preventive and treatment strategies to fight the impact of delirium and DSD on the health of older adults.

## Supporting information

S1 STROBE Checklist Strengthening the Reporting of Observational Studies in Epidemiology (STROBE) checklist. (DOCX)

## Abbreviations

CAM: Confusion Assessment Method
CDR: Clinical Dementia Rating
DSD: delirium superimposed on dementia
GFR: glomerular filtration rate
HR: hazard ratio
IQCODE: Informant Questionnaire on Cognitive Decline in the Elderly
IQR: interquartile range
