# Supplementary material for: Association between delirium superimposed on dementia and mortality in hospitalized older adults: A prospective cohort study
Source: PLoS Med. 2017 Mar 28;14(3):e1002264. doi: 10.1371/journal.pmed.1002264 (PMC5370103; doi:10.1371/journal.pmed.1002264)
Supplement: S1 STROBE Checklist — (DOCX) [file pmed.1002264.s001.docx]

STROBE Statement—checklist of items that should be included in reports of observational studies

**Prognostic Effect of Delirium Superimposed on Dementia in Hospitalized Older Adults: A Prospective Cohort Study.**

|  | Item No | Recommendation |
| --- | --- | --- |
| **Title and abstract** | 1 | (*a*) Indicate the study’s design with a commonly used term in the title or the abstract: **title page.** |
|  |  | (*b*) Provide in the abstract an informative and balanced summary of what was done and what was found: **ABSTRACT,** **methods and findings section.** |
| Introduction | | |
| Background/rationale | 2 | Explain the scientific background and rationale for the investigation being reported: **INTRODUCTION, paragraphs 1-3.** |
| Objectives | 3 | State specific objectives, including any prespecified hypotheses: **INTRODUCTION, paragraph 3.** |
| Methods | | |
| Study design | 4 | Present key elements of study design early in the paper: **METHODS, paragraph 1.** |
| Setting | 5 | Describe the setting, locations, and relevant dates, including periods of recruitment, exposure, follow-up, and data collection: **METHODS, paragraph 1.** |
| Participants | 6 | (*a*) *Cohort study*—Give the eligibility criteria, and the sources and METHODS of selection of participants. Describe methods of follow-up: **METHODS, paragraph 2.** |
|  |  | (*b*) *Cohort study*—For matched studies, give matching criteria and number of exposed and unexposed: **not applicable.** |
| Variables | 7 | Clearly define all outcomes, exposures, predictors, potential confounders, and effect modifiers. Give diagnostic criteria, if applicable: **METHODS, paragraphs 3-4.** |
| Data sources/ measurement | 8* | For each variable of interest, give sources of data and details of methods of assessment (measurement). Describe comparability of assessment methods if there is more than one group: **METHODS, paragraphs 5-7.** |
| Bias | 9 | Describe any efforts to address potential sources of bias: **METHODS, paragraph 8; DISCUSSION, paragraphs 8-11.** |
| Study size | 10 | Explain how the study size was arrived at: **METHODS, paragraph 2.** |
| Quantitative variables | 11 | Explain how quantitative variables were handled in the analyses. If applicable, describe which groupings were chosen and why: **METHODS, paragraph 8.** |
| Statistical methods | 12 | (*a*) Describe all statistical methods, including those used to control for confounding: **METHODS, paragraphs 8-9.** |
|  |  | (*b*) Describe any methods used to examine subgroups and interactions: **not applicable.** |
|  |  | (*c*) Explain how missing data were addressed: **METHODS, paragraph 2.** |
|  |  | (*d*) *Cohort study*—If applicable, explain how loss to follow-up was addressed: **METHODS, paragraph 4.** |
|  |  | (*e*) Describe any sensitivity analyses: **METHODS, paragraph 9.** |

Continued on next page

| Results | | |
| --- | --- | --- |
| Participants | 13* | (a) Report numbers of individuals at each stage of study—eg numbers potentially eligible, examined for eligibility, confirmed eligible, included in the study, completing follow-up, and analysed: **RESULTS, Fig 1.** |
|  |  | (b) Give reasons for non-participation at each stage: **RESULTS, Fig 1.** |
|  |  | (c) Consider use of a flow diagram: **RESULTS, Fig 1.** |
| Descriptive data | 14* | (a) Give characteristics of study participants (eg demographic, clinical, social) and information on exposures and potential confounders: **RESULTS, Table 1.** |
|  |  | (b) Indicate number of participants with missing data for each variable of interest: **RESULTS, Fig 1.** |
|  |  | (c) *Cohort study*—Summarise follow-up time (eg, average and total amount): **METHODS, paragraph 4; RESULTS, paragraphs 1 and 3.** |
| Outcome data | 15* | *Cohort study*—Report numbers of outcome events or summary measures over time: **RESULTS, paragraphs 2-3, Fig 2, Tables 2 and 3.** |
| Main results | 16 | (*a*) Give unadjusted estimates and, if applicable, confounder-adjusted estimates and their precision (eg, 95% confidence interval). Make clear which confounders were adjusted for and why they were included: **RESULTS, Tables 2 and 3.** |
|  |  | (*b*) Report category boundaries when continuous variables were categorized: **RESULTS, Tables 2 and 3.** |
|  |  | (*c*) If relevant, consider translating estimates of relative risk into absolute risk for a meaningful time period: **RESULTS, Tables 2 and 3.** |
| Other analyses | 17 | Report other analyses done—eg analyses of subgroups and interactions, and sensitivity analyses: **RESULTS, paragraph 6.** |
| Discussion | | |
| Key results | 18 | Summarise key results with reference to study objectives: **DISCUSSION, paragraph 1.** |
| Limitations | 19 | Discuss limitations of the study, taking into account sources of potential bias or imprecision. Discuss both direction and magnitude of any potential bias: **DISCUSSION, paragraphs 8-10.** |
| Interpretation | 20 | Give a cautious overall interpretation of results considering objectives, limitations, multiplicity of analyses, results from similar studies, and other relevant evidence: **DISCUSSION, paragraphs 2-12.** |
| Generalisability | 21 | Discuss the generalisability (external validity) of the study results: **DISCUSSION, paragraph 8.** |
| Other information | | |
| Funding | 22 | Give the source of funding and the role of the funders for the present study and, if applicable, for the original study on which the present article is based: **Financial disclosure.** |
